# Danger ahead: the burden of diseases, injuries, and risk factors in the Eastern Mediterranean Region, 1990–2015

**DOI:** 10.1007/s00038-017-1017-y

**Published:** 2017-08-03

**Authors:** GBD 2015 Eastern Mediterranean Region Collaborators, Charbel El Bcheraoui, Raghid Charara, Ibrahim Khalil, Maziar Moradi-Lakeh, Ashkan Afshin, Michael Collison, Farah Daoud, Kristopher J. Krohn, Adrienne Chew, Stan Biryukov, Daniel Casey, Kelly Cercy, Fiona J. Charlson, Leslie Cornaby, Daniel Dicker, Holly E. Erskine, Alize J. Ferrari, Christina Fitzmaurice, Kyle J. Foreman, Maya Fraser, Joseph Frostad, William W. Godwin, Max Griswold, Nicholas J. Kassebaum, Laura Kemmer, Michael Kutz, Hmwe H. Kyu, Janni Leung, Patrick Liu, Joseph Mikesell, Grant Nguyen, Helen E. Olsen, Robert Reiner, Marissa Reitsma, Gregory Roth, Damian Santomauro, Alison Smith, Jeffrey D. Stanaway, Patrick Sur, Haidong Wang, Harvey A. Whiteford, Rima Afifi, Aliasghar Ahmad Kiadaliri, Alireza Ahmadi, Hamid Ahmadieh, Khurshid Alam, Noore Alam, Raghib Ali, Reza Alizadeh-Navaei, Rajaa Al-Raddadi, Khalid A. Altirkawi, Nahla Anber, Hossein Ansari, Palwasha Anwari, Hamid Asayesh, Solomon W. Asgedom, Tesfay Mehari Atey, Umar Bacha, Shahrzad Bazargan-Hejazi, Neeraj Bedi, Zulfiqar A. Bhutta, Donal Bisanzio, Zahid A. Butt, Amare Deribew, Shirin Djalalinia, Babak Eshrati, Alireza Esteghamati, Maryam S. Farvid, Farshad Farzadfar, Seyed-Mohammad Fereshtehnejad, Florian Fischer, Tsegaye T. Gebrehiwot, Nima Hafezi-Nejad, Randah R. Hamadeh, Samer Hamidi, Peter J. Hotez, Mohamed Hsairi, Jost B. Jonas, Amir Kasaeian, Yousef S. Khader, Ejaz A. Khan, Gulfaraz Khan, Abdullah T. A. Khoja, Tawfik A. M. Khoja, Jagdish Khubchandani, Jacek A. Kopec, Heidi J. Larson, Raimundas Lunevicius, Hassan Magdy Abd El Razek, Mohammed Magdy Abd El Razek, Reza Majdzadeh, Azeem Majeed, Reza Malekzadeh, Ziad A. Memish, George A. Mensah, Jamal T. Nasher, Carla M. Obermeyer, Farshad Pourmalek, Mostafa Qorbani, Amir Radfar, Anwar Rafay, Vafa Rahimi-Movaghar, Rajesh Kumar Rai, David L. Rawaf, Salman Rawaf, Amany H. Refaat, Satar Rezaei, Gholamreza Roshandel, Mahdi Safdarian, Sare Safi, Saeid Safiri, Mohammad Ali Sahraian, Payman Salamati, Abdallah M. Samy, Benn Sartorius, Sadaf G. Sepanlou, Masood A. Shaikh, Morteza Shamsizadeh, Badr H. A. Sobaih, Rizwan Suliankatchi Abdulkader, Arash Tehrani-Banihashemi, Mohamad-Hani Temsah, Abdullah S. Terkawi, Roman Topor-Madry, Olalekan A. Uthman, Stein Emil Vollset, Andrea Werdecker, Mohsen Yaghoubi, Mehdi Yaseri, Mustafa Z. Younis, Maysaa E. S. Zaki, Aisha O. Jumaan, Theo Vos, Mohsen Naghavi, Simon I. Hay, Christopher J. L. Murray, Ali H. Mokdad

**Affiliations:** Institute for Health Metrics and Evaluation, University of Washington, Seattle, WA USA

**Keywords:** Burden of disease, Eastern Mediterranean Region, Injuries, Risk factors, Disability-adjusted life years

## Abstract

**Objectives:**

The Eastern Mediterranean Region faces several health challenges at a difficult time with wars, unrest, and economic change.

**Methods:**

We used the Global Burden of Disease 2015 study to present the burden of diseases, injuries, and risk factors in the Eastern Mediterranean Region from 1990 to 2015.

**Results:**

Ischemic heart disease was the leading cause of death in the region in 2015, followed by cerebrovascular disease. Changes in total deaths ranged from a reduction of 25% for diarrheal diseases to an increase of about 42% for diabetes and tracheal, bronchus, and lung cancer. Collective violence and legal intervention increased by 850% during the time period. Diet was the leading risk factor for disability-adjusted life years (DALYs) for men compared to maternal malnutrition for females. Childhood undernutrition was the leading risk factor for DALYs in 1990 and 2005, but the second in 2015 after high blood pressure.

**Conclusions:**

Our study shows that the region is facing several health challenges and calls for global efforts to stabilise the region and to address the current and future burden of disease.

**Electronic supplementary material:**

The online version of this article (doi:10.1007/s00038-017-1017-y) contains supplementary material, which is available to authorized users.

## Introduction

The Eastern Mediterranean Region (EMR) is home to more than 500 million people, representing a diverse group of 22 countries: Afghanistan, Arab Republic of Egypt, Bahrain, Djibouti, Iraq, Islamic Republic of Iran, Jordan, Kingdom of Saudi Arabia (KSA), Kuwait, Lebanon, Libya, Morocco, Oman, Pakistan, Palestine, Qatar, Republic of Yemen, Somalia, Sudan, Syrian Arab Republic (Syria), Tunisia, and the United Arab Emirates (UAE). These countries have different gross domestic products, socio-demographic profiles, health indicators, and health system capacities and coverage (WHO EMRO 2017; Mandil et al. 2013). About 12.2% of the population comprises children under 5 years of age, and 20% are women of childbearing age (WHO EMRO 2013).

The region also has wide variation in per capita gross national product (GNP), ranging from a high of $134,420 in Qatar to a low of $2000 in Afghanistan (The World Bank GNI per capita 2017). While the Gulf States are some of the richest countries globally, poverty rates remain high in many other countries of the EMR. The proportion of the population living below the national poverty line, according to World Bank data, is more than 20% in seven EMR countries: Afghanistan (36%), Egypt (22%), Iraq (23%), Pakistan (22%), Palestine (22%), Sudan (47%), and Yemen (35%). In five of these countries, approximately one-third of the population is also food-insecure: Afghanistan (34%), Iraq (30%), Pakistan (30%), Sudan (33%), and Yemen (36%) (The World Bank Databank 2017).

This region faces several health challenges at a difficult time with wars, unrest, and economic changes (Mokdad et al. 2014, 2016). These events will put a strain on limited resources and impact the health gains achieved so far. In addition, the EMR has a large, young population, and current events will shape the well-being of future generations.

In this issue of the *Journal*, we report the burden of several diseases and risk factors in separate manuscripts: intentional injuries, lower respiratory infections, maternal mortality, mental health, obesity, vision loss, road traffic injuries, adolescent health, cancer, cardiovascular disease, child mortality, diabetes and chronic kidney disease, diarrhoea, and HIV (GBD 2015 EMR Diabetes and Chronic Kidney Disease Collaborators 2017e; GBD 2015 EMR Child Mortality Collaborators 2017d; GBD 2015 EMR HIV Collaborators 2017g; GBD 2015 EMR Diarrhea Disease Collaborators 2017f; GBD 2015 EMR Cancer Collaborators 2017c; GBD 2015 EMR Intentional Injuries Collaborators 2017h; GBD 2015 EMR Cardiovascular Disease Collaborators 2017b; GBD 2015 EMR Adolescent Health Collaborators 2017a; GBD 2015 EMR Lower Respiratory Infections Collaborators 2017i; GBD 2015 EMR Vision Loss Collaborators 2017n; GBD 2015 EMR Maternal Mortality Collaborators 2017j; GBD 2015 EMR Transportation Injuries Collaborators 2017m; GBD 2015 EMR Obesity Collaborators 2017l; GBD 2015 EMR Mental Disorders Collaborators 2017k). These topics were selected based on the burden of disease in the region as well as the interest of the collaborators and the scientific community. This manuscript provides the overall burden of diseases, injuries, and risk factors in the Eastern Mediterranean Region from 1990 to 2015 and provides an update of our previous publications (Mokdad et al. 2014, 2016; Khalil et al. 2016; Moradi-Lakeh et al. 2017a; Moradi-Lakeh et al. 2017b; Charara et al. 2017).

## Methods

### Overview

The Global Burden of Disease (GBD) 2015 methodology has been published elsewhere (Forouzanfar et al. 2016; Kassebaum et al. 2016a, b; Vos et al. 2016; Wang et al. 2016a, b). GBD 2015 uses a comprehensive approach to report causes of death with garbage code redistribution; a systematic and simultaneous estimation of disease incidence, prevalence, exposure to risks, and injuries; and statistical models to pool data, adjust for bias, and incorporate covariates. It uses several metrics to report results for health loss related to specific diseases, injuries, and risk factors: deaths and death rates, years of life lost due to premature mortality (YLLs), prevalence and prevalence rates for sequelae, years lived with disability (YLDs), and disability-adjusted life years (DALYs). It provides a comprehensive assessment of all-cause mortality and causes of death estimates due to 249 causes in 195 countries and territories from 1990 to 2015.

GBD estimates incidence and prevalence by age, sex, cause, year, and geography using a wide range of updated and standardised analytical procedures. GBD uses DisMod-MR, a Bayesian meta-regression tool first developed for GBD 2010 and GBD 2013 to determine prevalence and incidence by cause and sequelae.

GBD 2015 used the comparative risk assessment (CRA) framework developed for previous iterations of the GBD study to estimate attributable deaths, DALYs, and trends in exposure by age group, sex, year, and geography for 79 behavioural, environmental and occupational, and metabolic risks or clusters of risks over the period 1990–2015. Risk-outcome pairs were included in the GBD 2015 study if they met World Cancer Research Fund criteria for convincing or probable evidence. Relative risk estimates were extracted from published and unpublished randomised controlled trials, cohorts, and pooled cohorts. Risk exposures were estimated based on published studies, household surveys, census data, satellite data, and other sources. Two modelling approaches—a Bayesian meta-regression model and a spatiotemporal Gaussian process regression model—developed for the GBD study were used to pool data from different sources, adjust for bias in the data, and incorporate potential covariates. GBD uses the counterfactual scenario of theoretical minimum risk exposure level (TMREL) to attribute burden. TMREL is the level for a given risk exposure that could minimise risk at the population level. A summary exposure value (SEV) was developed for GBD 2015 as the relative risk-weighted prevalence of exposure. SEV ranges from zero when no excess risk exists in a population to one when the population is at the highest risk.

### Socio-demographic Index and decomposition of variance

GBD 2015 created a Socio-demographic Index based on lag-dependent income per capita, average educational attainment for ages 15 or older, and the total fertility rate. To analyse the drivers of change, GBD 2015 decomposed trends in diseases and attributable burden into contributions from population growth, change in population structure by age and sex, risk exposure, and risk-deleted cause-specific DALY rates.

GBD 2015 has four levels of causes that are mutually exclusive and exhaustive. Level 1 has three causes: communicable, maternal, neonatal, and nutritional disorders; non-communicable diseases; and injuries. Level 2 has 21 causes, while Levels 3 and 4 consist of disaggregated causes. GBD 2015 documented each step of the estimation processes, as well as data sources, in accordance with Guidelines for Accurate and Transparent Health Estimates Reporting (GATHER).

## Results

**Fig. 1:**
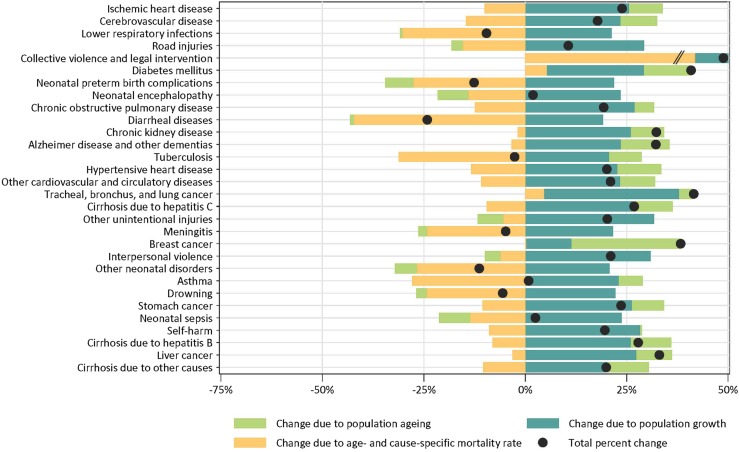
Eastern Mediterranean Region decomposition of changes in leading 30 causes of death due to population growth, population ageing, and changes in age-specific mortality rates, 2005–2015. Collective violence and legal intervention, which increased by 847%, was truncated for display purposes (Global Burden of Disease 2015 study, Eastern Mediterranean Region, 1990–2015)

Our results showed a major shift in burden of disease in the region and a wide variation by countries. Ischaemic heart disease (IHD) was the leading cause of death in the region in 2015, followed by cerebrovascular disease (Fig. 1). Among the leading 30 causes of deaths, there were variations in the drivers of changes in mortality from population growth, ageing, and changes to age-standardised rates of cause-specific mortality from 2005 to 2015. Changes in total deaths ranged from a reduction of 25% for diarrheal diseases to an increase of about 42% for diabetes and tracheal, bronchus, and lung cancer. Population growth accounted for increases across all causes, while population ageing led to increases in 18 causes. Declines attributable to changes in age-specific and cause-specific mortality rates varied markedly. Collective violence and legal intervention increased by 850% during the time period.

**Fig. 2:**
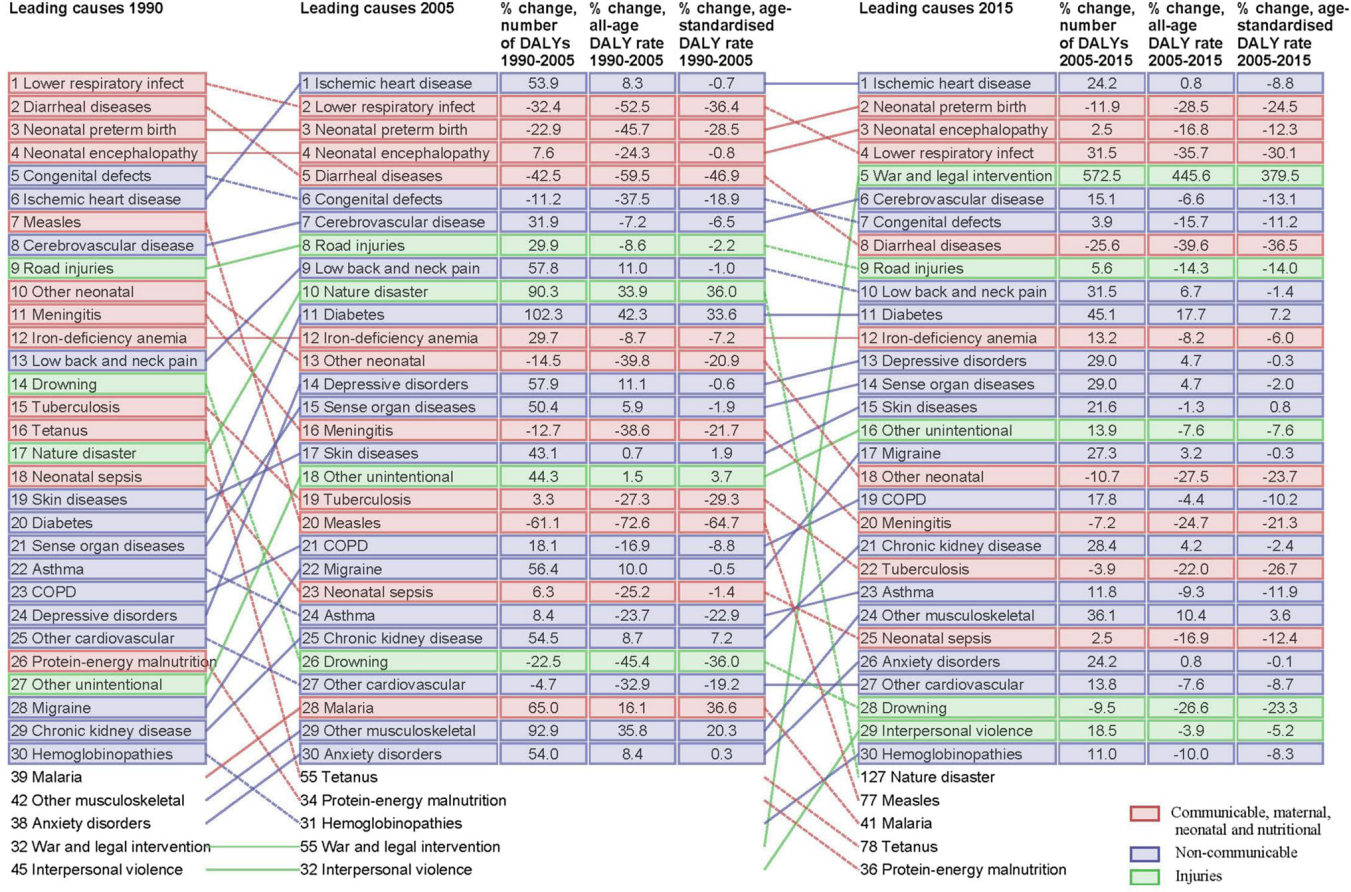
Leading 30 level 3 Eastern Mediterranean Region causes of disability-adjusted life-years (DALYs) for both sexes combined, 1990, 2005 and 2015. Causes are connected by arrows between time periods. Communicable, maternal, neonatal and nutritional causes are shown in red, non-communicable causes in blue and injuries in green. For the time period 1990–2005 and for 2005–2015, three measures of change are shown: percent change in the number of DALYs, percent change in the all-age DALY rate and percent change in the age-standardised DALY rate (Global Burden of Disease 2015 study, Eastern Mediterranean Region, 1990–2015)

Figure 2 shows the leading causes of disease burden over time in the EMR. Ischemic heart disease was the leading cause of DALYs followed by neonatal preterm birth complications, neonatal encephalopathy, lower respiratory infections, and war and legal intervention.

**Fig. 3:**
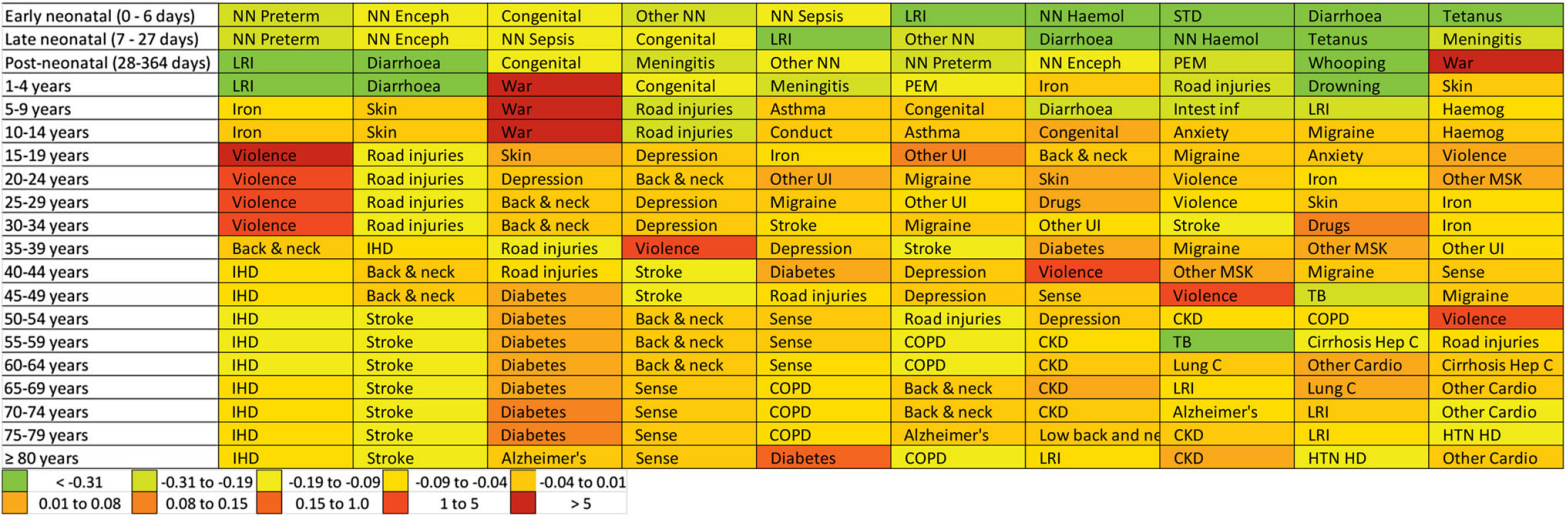
Leading ten Level 3 causes of Eastern Mediterranean Region age-specific disability-adjusted life-years (DALYs) in 2015. Each cause is coloured by the percentage change in age-specific DALY rate from 2005 to 2015. NN Preterm = neonatal preterm birth complications. NN Sepsis = neonatal sepsis and other neonatal infections. LRI = lower respiratory infections. Iron = iron-deficiency anaemia. HIV = HIV/AIDS. Back and neck = low back and neck pain. IHD = ischaemic heart disease. NN Enceph = neonatal encephalopathy due to birth asphyxia and trauma. Diarrhoea = diarrhoeal diseases. Skin = skin and subcutaneous diseases. Depression = depressive disorders. Stroke = cerebrovascular disease. Congenital = congenital anomalies. Diabetes = diabetes mellitus. COPD = chronic obstructive pulmonary disease. Alzheimer’s = Alzheimer’s disease and other dementias. PEM = protein-energy malnutrition. Conduct = conduct disorder. Sense = sense organ diseases. Other NN = other neonatal disorders. Intest inf = intestinal infectious diseases. Violence = interpersonal violence. NN Haemol = haemolytic disease and other neonatal jaundice. Anxiety = anxiety disorders. TB = tuberculosis. Lung C = lung, bronchial, and tracheal cancers. STD = sexually transmitted diseases excluding HIV. Haemog = haemoglobinopathies and haemolytic anaemias. CKD = chronic kidney disease. Other MSK = other musculoskeletal disorders. Drugs = drug use disorders. HTN HD = hypertensive heart disease. Whooping = whooping cough. Other UI = other unintentional injuries. War = collective violence and legal intervention. Cirrhosis Hep C = Cirrhosis and other chronic liver diseases due to hepatitis C. Other Cardio = Other cardiovascular and circulatory diseases. GBD = Global Burden of Disease (Global Burden of Disease 2015 study, Eastern Mediterranean Countries, 2005–2015)

Figure 3 shows the changes in the leading causes of DALYs from 2005 to 2015 by age. Violence and war increased from an early age to 55 years old. Diabetes increased among ages 40 and older. There were declines in some infectious diseases among children under 5. IHD remained the leading cause of DALYs for ages 40 and older.

**Fig. 4:**
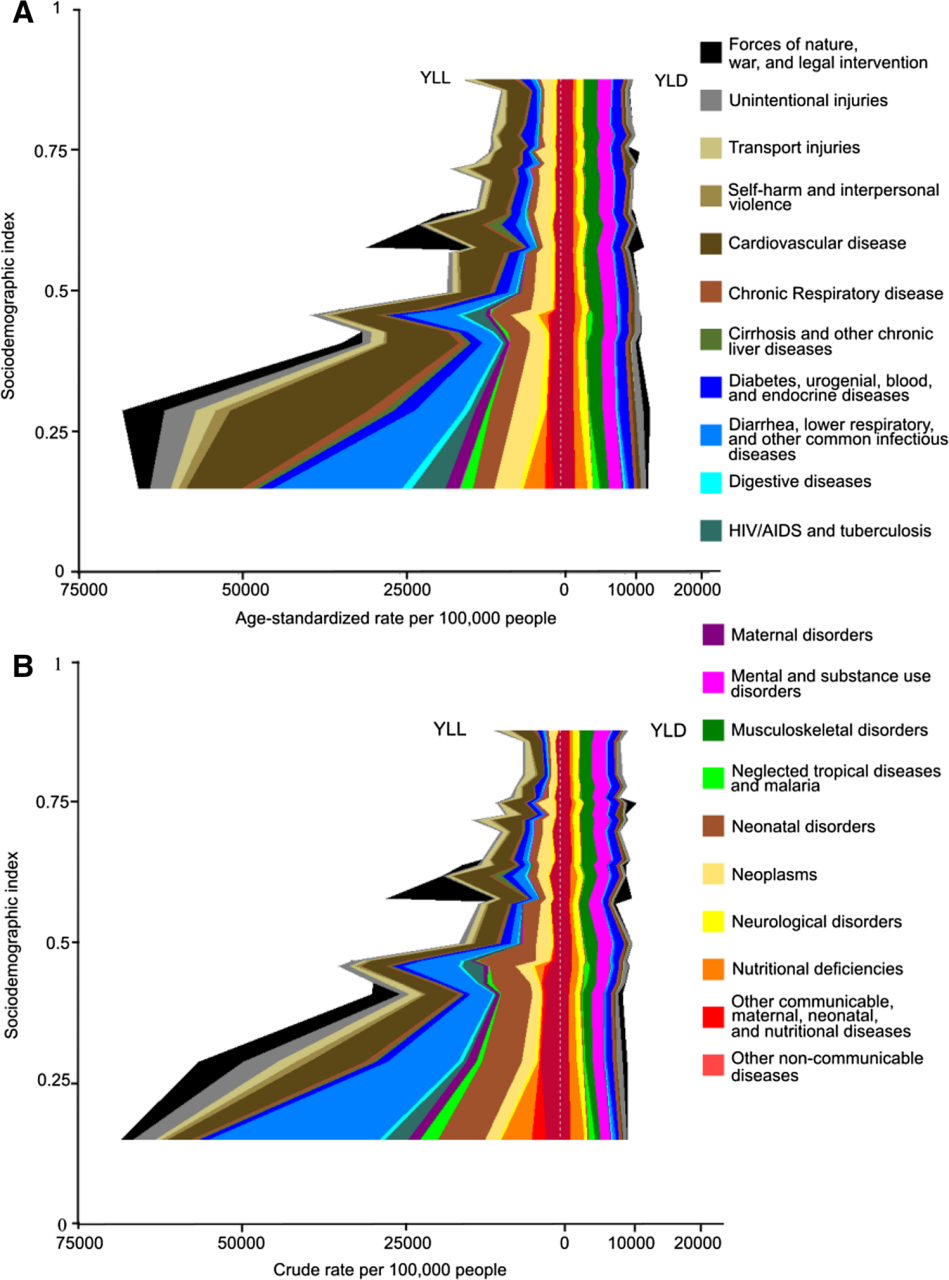
**a** Expected relationship between age-standardised years of life lost (YLL) and years lived with disability (YLD) rates per 100,000 and Sociodemographic index (SDI) and **b** all-age YLL and YLD rates (per 100,000) and SDI for 21 GBD Level 2 causes. These *stacked curves* represent the average relationship between SDI and each cause observed across the 22 Eastern Mediterranean Region countries in 2015. In each figure, the *y*-axis goes from lowest SDI to highest SDI. The *left side* shows rates for YLLs and the *right side* shows rates for YLDs; higher rates are further from the *midline*. The difference between (**a**) and (**b**) is the effect of shifts in population age structure expected with SDI. GBD = Global Burden of Disease (Global Burden of Disease 2015 study, Eastern Mediterranean Countries, 1990–2015)

Figure 4 shows the expected relationship between age-standardised and crude YLL and YLD rates for the region from 1990 to 2015 for Level 2 causes. Expected age-standardised YLL rates for infectious diseases declined with increased SDI. Cardiovascular disease (CVD) age-standardised YLL rates also declined with increased SDI. At the same time, age-standardised YLD rates for the top causes did not change much with SDI. At the higher SDI levels, YLD rates were the same as or higher than YLL rates. The spikes that appear at the left side of the figure show the impact of conflict and war. Increases are seen in YLLs from causes like war and injuries, as expected, but also from other types of causes, underscoring the effects these conflicts have on health systems when they occur.

**Fig. 5:**
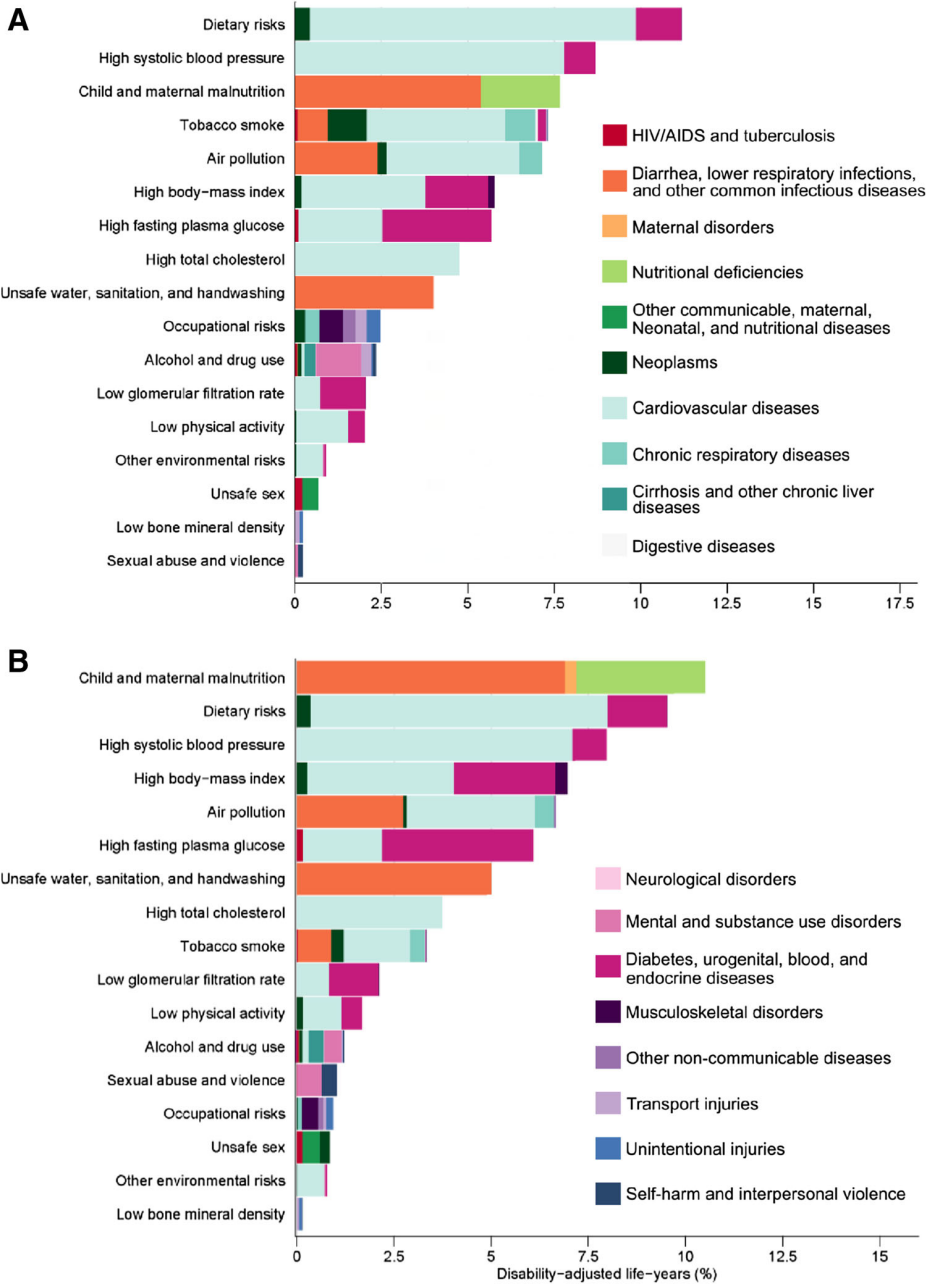
Eastern Mediterranean Region disability-adjusted life-years (DALYs) attributable to Level 2 risk factors for (**a**) men and (**b**) women in 2015 (Global Burden of Disease 2015 study, Eastern Mediterranean Countries, 1990–2015)

Figure 5 shows the EMR DALYs attributable to Level 2 risk factors for men and women in 2015. Diet is the leading risk factor for men, followed by high systolic blood pressure. Most of the DALYs burden for men is due to cardiovascular diseases and diabetes. Child and maternal malnutrition was the leading risk factor for DALYs for females, followed by diet. Child and maternal malnutrition impacted diarrhoea, lower respiratory infections, and nutritional deficiencies, while diet impacted CVD and diabetes.

**Fig. 6:**
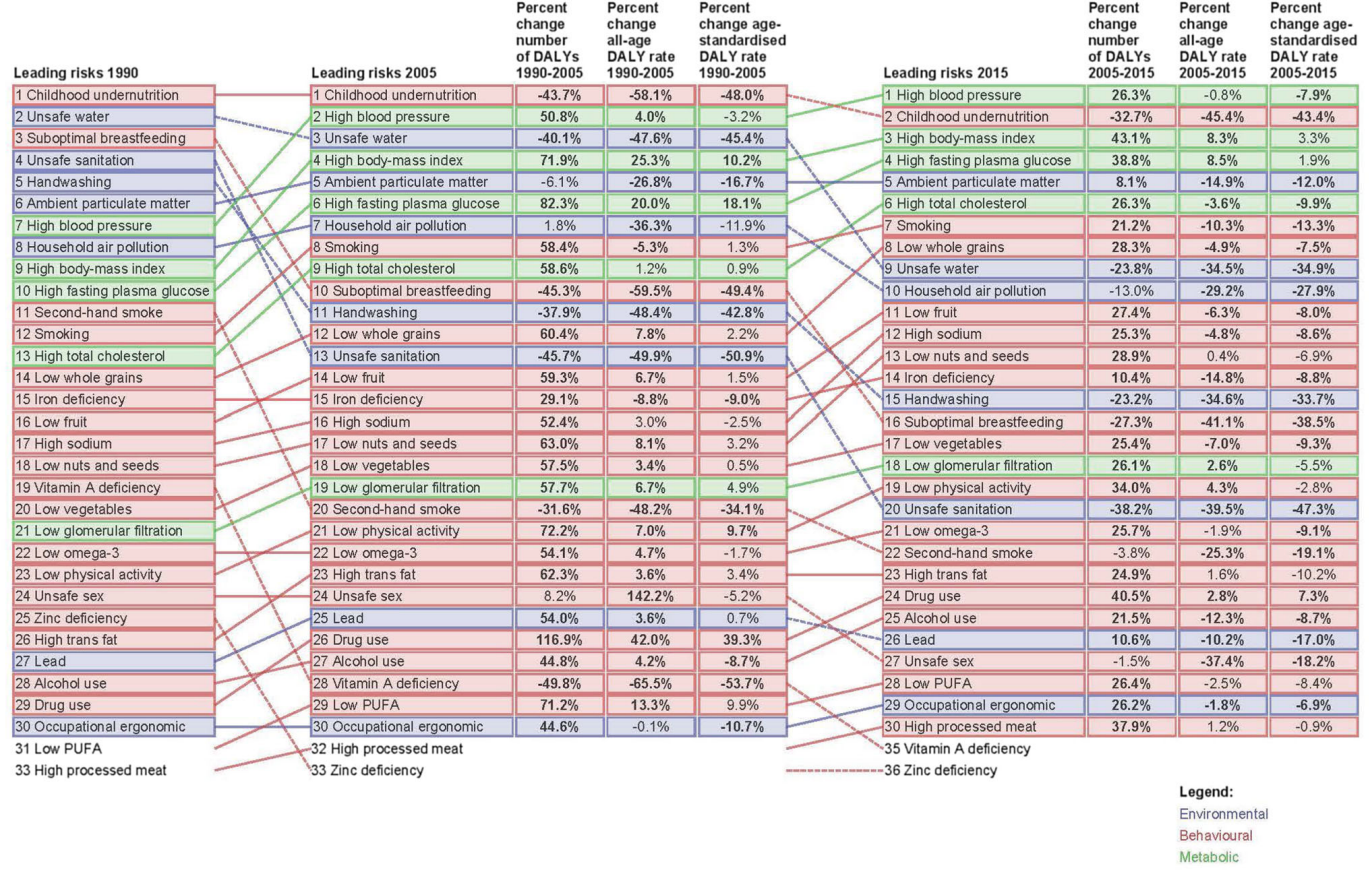
Leading 30 level 3 Eastern Mediterranean Region risk factors for disability-adjusted life-years (DALYs) for both sexes combined, 1990, 2005, and 2015. Risks are connected by arrows between time periods. Behavioural risk factors are shown in red, environmental risks in *blue* and metabolic risks in *green*. For the time period 1990–2005 and for 2005–2015, three measures of change are shown: percent change in the number of DALYs, percent change in the all-age DALY rate and percent change in the age-standardised DALY rate. Statistically significant increases or decreases are shown in *bold* (*p* < 0.05) (Global Burden of Disease 2015 study, Eastern Mediterranean Region, 1990–2015)

Figure 6 shows the EMR DALYs attributable to Level 3 risk factors and their changes from 1990 to 2005 and 2005 to 2015. Childhood undernutrition was the leading risk factor for DALYs in 1990 and 2005, but the second-leading in 2015 after high blood pressure. The percent change in the age-standardised DALY rate from 1990 to 2005 was a decline of 48%, compared to a decline of 43.4% from 2005 to 2015. Both obesity and high fasting plasma glucose increased from 1990 to 2005 and from 2005 to 2015, but the rate of increase was slower from 2005 to 2015.

e-Figure 1 shows the decomposition of changes for all-cause DALYs to Level 3 risk factors from 1990 to 2015 for the region. Overall changes in in all causes of DALYs ranged from a decline of 75% to an increase of a little over 200%. Population growth contributed to the increase in DALYs for all risk factors, while population ageing contributed to an increase for 33 causes. Drug use had the highest increase in risk exposure, followed by high body mass index and high fasting plasma glucose. Changes in the risk-deleted DALYs rate resulted in a decline in all but six causes.

## Discussion

Our study shows that the region is facing several health challenges in addition to the impact of the ongoing wars and unrest. The region is dealing with an epidemiological shift in burden from infectious to chronic diseases. However, the recent events may lead to a resurgence of some communicable diseases that were declining before these events. Moreover, countries will have a strain on their efforts to control and prevent non-communicable diseases. Our findings call for global efforts to stabilise the region and to address the current and future burden of disease.

In addition, but also linked to other effects of unrest, several risk factors affecting health are present. Efforts to reduce and prevent these risk factors in the region should be a health priority. For example, poor diet is the leading cause of DALYs in the region. Many countries in the region are suffering from malnutrition and at the same time from poor diet that is leading to disease. Tobacco smoking and systolic blood pressure are among the top causes of DALYs. Some countries in the region need to enforce regulations on tobacco to control and prevent smoking initiation. Blood pressure medication is now cheap and affordable for many in the EMR, but this may not be true for some low-income countries in the region. However, mechanisms for early detection and proper management of high blood pressure should be adopted to reduce this burden. Viral hepatitis accounts for a large burden in the region, especially in Somalia, Pakistan, Djibouti, Afghanistan, and Egypt (Institute for Health Metrics and Evaluation 2016). The burden of hepatitis requires efforts to prevent the spread of the disease through minimising risk factors and providing proper immunizations. Moreover, screening and treatment for hepatitis C should be encouraged.

The EMR has a large burden from ambient air pollution. Ambient air pollution is associated with increased mortality and morbidity (WHO 2005). Our study showed that ambient particulate matters are the 5th leading DALYs risk. We have previously reported on the global rise in the burden of air pollution (Cohen et al. 2017).

Several countries in the region face a major environmental challenge due to lack of water, rising temperatures, and sand storms. Our findings call for renewed efforts to address the burden of ambient air pollution. Indeed, unlike other risk factors or challenges faced by the region, environmental health requires strong governmental commitments to implement the global environmental standards and utilise the currently available technologies to reduce the burden.

The wars in the region, especially in Yemen, Iraq, and Syria, are taking a large toll on the health of the population. The immediate impact of the wars has been very high, with increased mortality due to violence. Moreover, these events will lead to increased health burden in the future as the next generation in many countries in the region is being raised under the harsh conditions of malnutrition and lack of preventive health services.

The wars and unrest have led to major migration and a large refugee population inside and outside the region. For many host countries, the existing health systems and infrastructure do not support such a large additional population. In Lebanon, for example, public schools are providing education to Lebanese and Syrian children, but the public school infrastructure is not capable of dealing with such a large number of students. This has resulted in a double shift in schools and put a large strain on the system. The same applies to other services besides health, and in other countries.

Countries in the region need to continue to strive to achieve universal health coverage, strong screening and prevention programs, and effective health delivery systems. The countries in the region can also learn from the systems put in place for the training and accreditation of health professionals, priority-setting, and the implementation of evidence-based health care undertaken by some other developed countries. Investment in health systems can create jobs and improve economic growth, in addition to the direct benefits on health outcomes. It is also important to look at the wider determinants of health—such as poverty, housing, education, and employment; and to empower women to have a dramatic effect on health outcomes.

Despite the market failures, the private sector can still play an important role in regional health systems. Providing an amiable environment to foster competition between public and private providers will ensure better quality and efficiency of services delivered. Better engagement of the private providers can reduce the burden of financing on the public sector. In this respect, movement from input-based payment toward a performance-based payment system is urgently needed. Furthermore, considering the variety of health challenges facing the region, it is vital for the countries to adopt concepts of health in all policies. This can be achieved by developing a national body that focuses on setting up collaborative efforts among all sectors to incorporate health issues into all policy areas aiming to promote, protect, preserve, and restore population health.

A critical component to improve current and future health in the EMR is the ability to effectively and efficiently diagnose the challenges to health and well-being faced by the region. The Global Burden of Disease offers accurate and comprehensive information on the global burden of diseases, injuries, and risk factors, and develops new analytic methods and data visualisation tools to support the understanding of this information and to empower policymakers and health leaders to act. However, the region still has a long way to go in terms of having adequate and timely data to better inform decision-makers of the burden. Therefore, there is an urgent need to improve vital statistics, data sources, and surveillance systems in the region to better serve their purpose.

The region is in dire need of a comprehensive plan to build on existing expertise and projects to address the health challenges that exist at the nexus of human health, environmental resilience, and social and economic equity. The region does not have proper health translation and implementation efforts to address its growing health challenges. Unfortunately, many countries have focused on curative rather than preventive systems. Indeed, this will limit the pace of progress needed to address many of the emerging challenges such as non-communicable diseases and the emergence of infectious diseases in countries with wars and unrest. This lack of progress is evidenced by wide health disparities between and within countries and exists despite the identified organisations and forums that offer recommendations for intervention, such as the World Health Organization and others.

This comprehensive plan needs to review and compile information on prior health interventions for each targeted topical area of burden from peer-reviewed and grey literature and include both successful and negative outcomes (as much can be learned from failures as from successes), as well as potential unintended consequences of interventions. The plan should include a synthesis of the available quantitative and qualitative evidence on interventions and innovations to develop a summary of why specific work around a risk or disease succeeds or fails. This analysis will develop a deeper understanding of the necessary ingredients for success (i.e., to identify underlying social, economic, legal, and public policy features). This will allow health actors to design and conduct innovative research on intervention effectiveness, implementation, scale-up, dissemination, and economic return in partnership with community, governments, foundations, and other collaborators. This work should draw on resources including, but not limited to, the United Nations’ Sustainable Development Goals, the Disease Control Priorities publications, and the World Health Organization’s “Best Buys”.

Health education and training are crucial to improve the burden of disease in the EMR. There is a dire need for opportunities and funding to offer training for public officials (e.g., health ministers, policymakers, and local health officers) and program leaders, provided both on-site and on a regional scale at in-country sites in collaboration with other countries. These trainings should provide participants with actual experience implementing the interventions that have been developed. Finally, there is a need to scale up the public health workforce across the region, to ensure that the right policies are developed, implemented, and enforced.

Health advocacy and effective program and policy dissemination must be at the forefront of all health activities. The region needs a catalyst for change at both country and regional levels by providing a platform on which local and global strategies and successes are collaboratively shared among local communities and countries. This, in turn, will encourage adoption, successful implementation, and ultimately, sustainability of population health.

The future of health in the region is grim unless the wars and unrest stop. Regional health professionals are dealing with overwhelming challenges and can barely meet basic health needs. The best intervention for a better future is an international plan to stabilise the region. All countries have an equally important role to play in bringing an end to the unrest and starting to rebuild.

Our study has some limitations. The availability and quality of data for some countries in the region pose substantial challenges for cause of death analysis. Many countries in the region do not have strong vital registration systems. Our GBD methodology makes extensive efforts to reduce the effects of variable data quality, and we have used standardised methods for each cause that are the same for all countries. We also provide uncertainty intervals for each of our estimates that take into account the data issues, and we provide all our data sources and show what is available for every country on our website (Institute for Health Metrics and Evaluation 2017). Our web visualisations allow comparison of raw data to final estimates and show the impact of our models and methods of dealing with data quality or lack of it. Finally, our study provides the national burden and hence masks large disparities within a country.

### Conclusion

Our study shows a tremendous impact of war and violence on the health of the region. The results show that in recent years, many of the health gains for some countries have slowed and several health conditions that were under control are re-emerging. These findings clearly indicate that the future health of the region is in danger. Immediate efforts to stabilise the region and improve the health of the population are urgently needed.

## Electronic supplementary material

Below is the link to the electronic supplementary material.

Supplementary material 1 (XLSX 24 kb)

Supplementary material 2 (DOCX 66 kb)
