# Supplementary material for: Danger ahead: the burden of diseases, injuries, and risk factors in the Eastern Mediterranean Region, 1990–2015
Source: Int J Public Health. 2017 Aug 3;63(Suppl 1):11–23. doi: 10.1007/s00038-017-1017-y (PMC5973982; doi:10.1007/s00038-017-1017-y)

Electronic Supplementary Material

**Article title:**

Danger ahead: The burden of diseases, injuries, and risk factors in the Eastern Mediterranean Region, 1990–2015

**Journal:**

International Journal of Public Health

**Authors:**

GBD 2015 Eastern Mediterranean Region Collaborators

**Corresponding author:**

Ali H. Mokdad

Institute for Health Metrics and Evaluation, University of Washington, Seattle, WA, United States

Email: mokdaa@uw.edu

e-Figure 1. Eastern Mediterranean Region decomposition of changes in all-cause disability-adjusted life-years(DALYs) attributable to level 3 risk factors from 1990 to 2015 due to population growth, population ageing, risk exposure and the risk-deleted DALY rate. Risks are reported in order of percent change in the number of attributable DALYs from 1990 to 2015. This figure excludes DALYs attributable to unsafe sex because it is not estimated based on exposure and relative risk. (Global Burden of Disease 2015 study, Eastern Mediterranean Region, 1990-2015)


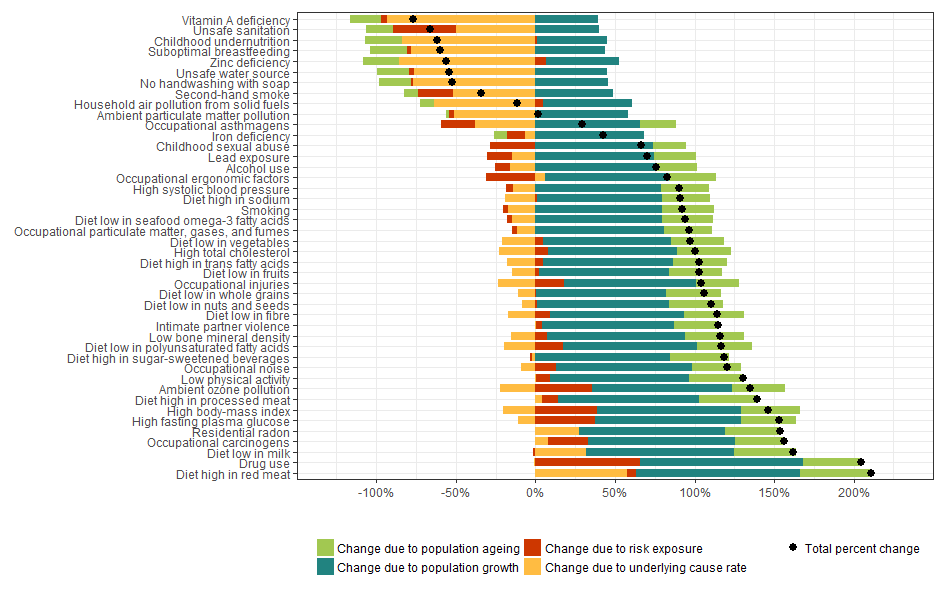

Supplement: Supplementary file 2 — Supplementary material 2 (DOCX 66 kb) [file 38_2017_1017_MOESM2_ESM.docx]
